# Current Evidence-Based Interdisciplinary Treatment Options for Pediatric Musculoskeletal Pain

**DOI:** 10.1007/s40674-018-0101-7

**Published:** 2018-06-14

**Authors:** Line Caes, Emma Fisher, Jacqui Clinch, Christopher Eccleston

**Affiliations:** 1 Division of Psychology, Faculty of Natural Sciences, University of Stirling, Stirling, FK9 4LA UK; 2 Centre for Pain Research, University of Bath, Bath, UK; 3 Bristol Royal Children’s Hospital, University of Bristol and Royal National Hospital for Rheumatic Diseases, Bath, UK; 4 Department of Experimental, Clinical and Health Psychology, Faculty of Psychology and Educational Sciences, Ghent University, Ghent, Belgium

**Keywords:** Pediatric chronic musculoskeletal pain, Interdisciplinary care, Lifestyle counseling, Complementary and alternative medicine, Digital delivery

## Abstract

**Purpose of review:**

We review the prevalence of pediatric chronic musculoskeletal pain, the clinical need, the evidence for pharmacological, psychological, physical and, complementary approaches to pain management, and the possible future development of interdisciplinary and distance care.

**Recent findings:**

We summarize the Cochrane Systematic Reviews on pharmacological interventions, which show a lack of evidence to support or refute the use of all classes of medication for the management of pain. The trials for NSAIDs did not show any superiority over comparators, nor did those of anti-depressants, and there are no trials for paracetamol, or of opioid medications. There are studies of psychological interventions which show promise and increasing support for physical therapy. The optimal approach remains an intensive interdisciplinary programmatic treatment, although this service is not available to most.

**Summary:**

1. Given the absence of evidence, a program of trials is now urgently required to establish the evidence base for analgesics that are widely prescribed for children and adolescents with chronic musculoskeletal pain. 2. Until that evidence becomes available, medicine review is an essential task in this population. 3. We need more examples and efficacy evaluations of intensive interdisciplinary interventions for chronic pain management, described in detail so that researchers and clinicians can unpack possible active treatment components. 4. Online treatments are likely to be critical in the future. We need to determine which aspects of treatment for which children and adolescents can be effectively delivered in this way, which will help reduce the burden of the large number of patients needing support from a small number of experts.

## Introduction

Chronic pain, defined as pain lasting for more than 3 months, is common amongst children and adolescents (also referred to as young people or youth), with prevalence of pediatric chronic pain ranging between 11 and 38% [1]. The most common types of chronic or persistent pain for young people include headaches, recurrent abdominal pain, and musculoskeletal pain [1]. Pediatric chronic musculoskeletal pain (PCMP), described as pain in the muscles, ligaments, tendons, and/or bones lasting for longer than 3 months, will be the focus of this article. This operationalization of PCMP includes conditions such as juvenile idiopathic arthritis (JIA) and fibromyalgia syndrome (FMS). Prevalence rates across studies vary widely; prevalence ranges between 8.5 and 40% for children and adolescents with chronic musculoskeletal pain, and findings are relatively consistent in reporting knee, back, and neck as the most common sites [1]. Prevalence, as well as disability due to PCMP, increases with age [1]. A recent population-based evaluation of the annual primary care consultation for pediatric musculoskeletal problems revealed that 8% of British children and adolescents present to primary care for musculoskeletal aches and pains at least once a year with an observed rise in consultations with age [2]. However, findings with respect to sex differences are mixed. While most prevalence studies report higher levels of musculoskeletal pain in girls [1], others report higher levels in boys [2, 3] and consultations for a musculoskeletal problem were more common amongst boys than girls [3]. Further research to clarify these inconsistencies in prevalence and consultation rates is needed but could be partially explained by the cause of musculoskeletal pain. For example, exercise- or trauma-related causes of the pain are more prevalent in boys compared to girls [2].

Chronic musculoskeletal pain can also accompany inflammatory, infective, and malignant musculoskeletal disease [4]. For instance, despite significant advances in medical treatments for children with JIA, persistent pain is a common complaint. Pain has been shown to be a primary determinant of the lower physical, emotional, and social functioning in these children [5]. The degree of disabling pain does not always mirror inflammatory joint activity, and a growing body of research highlights the importance of environmental and psychosocial influences on the pain experience of children, in addition to the contribution of disease activity [6, 7].

The experience of chronic musculoskeletal pain, irrespective of etiology, in childhood and adolescence has been consistently associated with lower health-related quality of life in domains such as physical health, psychological, emotional, social, and school functioning [8]. In particular, children and adolescents suffering from chronic musculoskeletal pain report lower physical functioning, attending school less often compared to their peers, reduced engagement with social and recreational activities, poorer sleep quality and fewer hours asleep, and mental health issues such as higher anxiety and depressive symptoms [9, 10]. The economic cost associated with the experienced disability due to pediatric chronic pain are high, with recent figures revealing a cost of $19.5 billion of chronic pain conditions alone (i.e., excluding pain associated with other long-term conditions which might also include pain as a symptom) [11]. Several psychosocial variables, such as parental responses to pain and catastrophic thinking [12], have been found to influence disability experienced due to PCMP, thereby highlighting the need and benefit of an interdisciplinary approach towards treatment and management. Furthermore, if untreated, children and adolescents with chronic pain are at risk of experiencing continued persistent pain and associated disability as an adult [1, 13, 14••]. Consequently, provision of adequate, evidence-based treatment for PCMP is crucial to offset a lifelong trajectory of disability [15].

In this review, we critically evaluate the evidence for an interdisciplinary approach towards treatment of PCMP, which mainly focuses on pharmacological, psychosocial, and physical support. Although evidence for other aspects of treatment, such as healthy diet, sleep hygiene, complementary, and alternative medicines is limited, their potential beneficial role within integrative care will be discussed. While each of the discussed treatment approaches can be successful when delivered in isolation, interdisciplinary care involving pharmacological, physical, psychological, and alternative therapies is a preferred treatment approach in this population [15, 16••].

## Treatment

### Pharmacologic treatment

Pharmacological treatments are usually the first form of treatment for PCMP. If there is an underlying disease process, then targeted treatments will be used to address this alongside pain management. Unfortunately, the current pharmacological treatments used to treat pain directly (including NSAIDs, opioids, and anti-depressants) in children and adolescents with pain and stiffness associated with their PCMP condition(s) are not as effective as one would hope. The number of analgesics and interventions used is a sign that there are no well-controlled therapeutic trials in pediatric chronic pain. While there are some ethical and practical challenges with establishing the efficacy in prospective, randomized controlled trials of pharmacological drugs in children and adolescents, the bigger challenge is to obtain the interest and support of pharmacological companies for such pediatric trials.

A recent Cochrane suite of reviews investigating the efficacy of pharmacological interventions for children and adolescents with chronic pain found seven randomized controlled trials delivering NSAIDs to youth with various forms of JIA, compared to active comparators [17•]. The NSAIDs delivered included aspirin, celecoxib, fenoprofen, ibuprofen, indomethacin, ketoprofen, meloxicam, naproxen, and rofecoxib. However, not all trials reported on outcomes of pain and functioning. For example, only three studies reported pain relief of 30% or greater, but due to the heterogeneity of samples and drugs delivered, results could not be combined into a singular meta-analysis. Further, when assessing each of the three studies individually, NSAIDs were not more beneficial at reducing pain intensity compared to the active comparators. Nevertheless, patient global impression of change (one study) and carer global impression of change (four studies) were mostly positive towards NSAIDs. There were a number of adverse events identified across the studies, including serious adverse events and withdrawals due to adverse events. The quality of evidence for all outcomes ranged from very low to low meaning there is very little confidence in the effect estimate and the true effect is likely to be substantially different from the estimate of effect. Overall, the review concluded that there was no evidence to suggest that NSAIDs were effective in treating chronic pain, including chronic musculoskeletal pain, in children and adolescents, or that one NSAID was more effective than another [17•].

In other Cochrane reviews, review authors did not find any studies eligible for inclusion to investigate the efficacy of paracetamol [18•] or opioids [19•] for children and adolescents with any chronic pain condition, including PCMP. Four studies (one study investigating children and adolescents with chronic widespread pain type 1, delivering gabapentin or amitriptyline) investigated the effects of anti-depressants in children and adolescents [20]. However, no studies reported on pain reduction or physical functioning. Similar to NSAIDs, there were adverse events, including serious adverse events and withdrawals due to adverse events. Further, quality of evidence was rated as very low.

Taken together, as described previously [21], the lack of evidence to support the efficacy of any of these therapies in children and adolescents is remarkable. Following these recent Cochrane reviews, a program of trials is now urgently required to establish the evidence base for analgesics that are still widely prescribed for PCMP. Until more evidence becomes available, and the potential for harm, it will be essential to pro-actively review the medicine intake and uptake within the context of PCMP. Polypharmacy (i.e., the risk of dosing and combination errors) and confusion over the utility of previously and currently prescribed medication is common [4] Consequently, drug withdrawal, detoxification, and replacement should be carefully managed [4].

### Psychological treatment

Psychological therapies typically aim to deliver psychoeducation, reduce pain intensity, increase functioning, and deliver adaptive coping skills to children and adolescents [22, 23]. Such interventions are frequently delivered in Western chronic pain clinics to children and adolescents with chronic musculoskeletal pain. The content of psychological therapies delivered for PCMP includes cognitive-behavioral therapy, coping skills training, and exposure and acceptance therapy.

Cognitive-behavioral therapy (CBT) is the most commonly delivered psychological therapy for pediatric chronic musculoskeletal pain conditions [24••, 25]. Psychoeducation, usually the first session of psychological interventions, explains the connection between thoughts, feelings, and behaviors. This is particularly important for children and adolescents with chronic musculoskeletal pain who report fatigue and tiredness, stiffness, and pain. Negative thoughts that pain is unmanageable and avoidance strategies of activities that may cause higher pain are challenged. Further the connection between low or negative mood and avoidance behaviors are explained. Therefore, coping strategies when experiencing pain spikes are discussed, so that adaptive behaviors can be practiced [24••]. Skills such as thought stopping/reframing, distraction, problem-solving, relaxation exercises (e.g., deep breathing, imagery), and pacing (covered in physical activity section) often make up the remaining session within CBT for PCMP. Some trials have also delivered psychological treatments to parents in addition to the child, which include providing parents with information and skills on adaptive coping skills, healthy lifestyles, and operant conditioning [26, 27].

Systematic reviews investigating the efficacy of psychological therapies for youth with any type of chronic pain produce small to moderate effects at reducing pain and disability immediately following treatment, and for up to 12 months following treatment [24••, 25, 29]. However, when combined in a meta-analysis, psychological treatments are not beneficial at reducing anxiety and depression symptomology for children and adolescents with chronic pain conditions. Typically, evidence for psychological interventions is very low or of low quality, meaning there is very little confidence in the estimate of effect [24••, 29]. In 2014, Fisher et al. analyzed chronic musculoskeletal pain as a pain-specific sub-group [25]. In this meta-analysis, one study investigated adolescents with JIA [30], two studies included children and adolescents with FMS [31, 32], and two studies included a mixed sample of chronic pain, which included children and adolescents with musculoskeletal pain [27, 28]. From this analysis, the authors found beneficial treatment effects for reducing pain intensity, disability, and depressive symptoms post-treatment, and for disability at follow-up [28]. Other outcome effects were not maintained. Only two studies contributed to the analysis of the impact on anxiety, and no beneficial effect was found. Similar to studies investigating other types of chronic pain conditions (e.g., headache, recurrent abdominal pain [33, 34]), no data were presented in trials on sleep outcomes and no conclusions can be made on whether psychological interventions can improve sleep, which is known to increase pain intensity and be disrupted in children and adolescents with pain [10].

Despite the evidence for CBT from these systematic reviews, pain also impacts other areas of functioning, not tackled by either treatment approaches, including school and friendships [35, 36]. Pilot trials have indicated that *peer support* can play an important role in supporting adolescents with JIA [37•]. In an 8-week peer-to-peer trial where adolescents with JIA interacted with young adults with JIA who had been trained in mentoring, all participants reported that they would recommend to their peers and reported that they were better able to manage their symptoms, in comparison to controls [37•]. Although there has been little investigation beyond this trial, social support interventions may represent an important area to investigate in the future.

### Physical therapy and exercise

Physical therapy and exercise is a key component of interdisciplinary treatment approaches for PCMP. However, systematic research evaluating the effectiveness within this particular population is scarce [16••]. Beyond educating families on the relevance and importance of regular physical activity engagement and building this into their child’s daily routing, physical therapy includes specific exercises aimed at increasing muscle strength and flexibility [38]. Examples of specific exercises include range of motion, stretching, and moderate aerobic exercises [38]. While evidence for physical therapy in adults with chronic musculoskeletal pain is strong [38], which lead to standard recommendations, evidence for PCMP is mixed and there are currently no standardized recommendations of exercise levels. For instance, in youth with juvenile FMS, exercise therapy has shown a positive impact on physical function, FMS symptoms, quality of life, and pain [39]. However, a systematic review of physical exercise for JIA reveals trends towards favorable short-term outcomes on increasing quality of life, functional ability, and aerobic capacity, but these effects were neither clinical nor statistically significant [40].

Adherence to the recommended exercise levels is a key challenge identified by clinicians, particularly for young people [38]. Not only can it be challenging to incorporate physical activity within a busy daily routine, engaging in physical activity can increase pain experiences, especially at the beginning of treatment, for children and adolescents suffering from PCMP [15]. To overcome this barrier, it is crucial to provide physical therapy within a cognitive-behavioral framework, with psychological support and a focus on pacing and goal setting [15, 16••; see more on goal setting within the section on psychological treatments]. Within this cognitive-behavioral framework, the patient sets his/her own goals for exercise engagements and plans to reach these goals within a realistic timeframe, in consultation with a physiotherapist and psychologist [15]. The evidence of the effectiveness of pacing interventions, for either adult or pediatric populations, is quite limited however. This lack of evidence is partly due to the absence of a generally agreed definition of pacing; a systematic review uncovered a wide range of definitions and operationalization of pacing within the context of chronic pain treatment. Definitions ranged from general coping techniques for chronic pain, to defining pacing as a planning strategy to conserve energy with the goal of increasing the level and range of activities [41]. The most widely used broad definition of pacing was put forward by Nielson et al. 2013: “*the regulation of activity level and/or rate in the service of an adaptive goal or goals*” [42, p., 465]. However, as illustrated by a recently formulated Activity Pacing Framework [43], the term activity pacing is too broad and is being used as an overarching label, to refer to subcomponents or to refer to a particular conceptualization. Such continued use of the term is confusing and untenable. The need for more clarity on the construct of pacing was highlighted in a recent meta-analysis revealing positive correlations between measures of pacing and avoidance [44]. A consensus on a definition, operationalization, and assessment of pacing within interdisciplinary treatment of PCMP will be crucial for appropriate evaluation of this key component of treatment.

### Other treatments

#### Lifestyle counseling

Accumulating evidence shows a strong association between weight increases, damage to the musculoskeletal structure, and pain complaints, which particularly impacts the ankles, feet, and knees [45]. Despite this link, to the best of our knowledge, no research is available on the impact of dietary changes aimed at managing PCMP. Nevertheless, several integral pain management and self-management programs do include skills aimed at promoting healthy lifestyle such as balanced diet and sleep hygiene for both children and their parents [30, 46]. For example, specific to children and adolescents with PCMP, healthy diets should ensure that calcium levels are appropriate, to facilitate the development of strong and healthy bones. In some occasions, particularly during winter months when Vitamin D deficiency is common, calcium levels are monitored, and Vitamin D supplements need to be considered to maintain optimum calcium levels [46]. However, only weak evidence is available to support the benefits of Vitamin D in managing chronic pain conditions and this evidence is limited to adult populations [47].

Further, given the bidirectional associations between musculoskeletal pain and sleep [10], lifestyle counseling for PCMP conditions often addresses the critical role of appropriate sleep hygiene [30, 46]. However, there has been little investigation into the treatment effects for sleep hygiene in children and adolescents with chronic pain in general or PCMP in particular [25]. In a randomized controlled trial that compared remotely delivering cognitive-behavioral therapy to educational control, authors did not find any post-treatment effects of sleep quality between groups [26]. Nevertheless, therapies that treat insomnia in children and adolescents (without chronic pain) are efficacious, and therefore there is promise in adapting these treatments successfully for PMCP [48].

#### Occupational therapy

Given the substantial impact of musculoskeletal pain on daily activities, an interdisciplinary approach towards managing PCMP often includes occupational therapy. Occupational therapy aims to increase engagement with daily life tasks despite the presence of PCMP. The implementation of occupational therapy varies widely and typically includes one or more of the following approaches: training of motor function, training of skills, instructions on joint protection, counseling on balancing daily life activities, and/or advice and instruction in the use of assistive devices and provision of splints [49]. While a systematic review provides evidence for benefits of comprehensive occupational therapy as well as instruction on joint protection and provision of splints on improving pain and functional ability in adults with rheumatoid arthritis [49], no studies evaluated the benefits of occupational therapy approaches for children and adolescents with musculoskeletal pain could be identified.

#### Complementary and alternative medicine

The inclusion of complementary and alternative medicine (CAM) approaches as part of interdisciplinary treatment for pediatric chronic pain has increased substantially in the last decade [50], with children and adolescents suffering from chronic musculoskeletal pain being amongst the highest users of CAM [51]. CAM is a “*group of diverse medical and healthcare approaches that are not presently considered part of conventional medicine*” [50, p., 453], with approaches within pediatric chronic pain including hypnosis, massage, acupuncture, art and music therapy, herbal medicines, and yoga [51]. The implementation and popularity of these CAM approaches are growing, yet evidence on their effectiveness for improving PCMP remains scarce and there are some current challenges with evaluating these interventions.

For example, a small number of studies reveal positive attitudes towards *acupuncture* (on its own or combined with *hypnotherapy*) as a pain-relieving strategy by children and adolescents with chronic pain, including PCMP. While these positive attitudes support the feasibility and acceptability of acupuncture [52], there is limited objective evidence for the usage, effectiveness, and cost-effectiveness of acupuncture within treatment of PCMP [53]. Similarly, although, *massage techniques* are frequently adopted to relief PCMP, a key problem with evaluating the effectiveness is the range of implementation of these massage techniques from delivery by a licensed massage therapist [54] to daily parent-delivered massage [52]. Despite this, there is preliminary evidence for the benefit of massage techniques in reducing pain, distress, stiffness, tension, and discomfort [52, 54]. Finally, *yoga* is a treatment approach that is gaining in popularity amongst youth. One pilot feasibility trial in youth with rheumatoid arthritis found notable improvements of medium to large beneficial effect sizes in reducing pain, pain disability, mental health, and increasing vitality and self-efficacy amongst participating youth [55]. However, qualitative reports from participants further confirm the beneficial impact of yoga on the child’s well-being, but quantitative reports did not indicate reduced pain intensity. Qualitative data also revealed a lack of continuing with yoga after the short 6-week program, with an associated diminishing impact of the immediate benefits [55]. Consequently, preliminary evidence for many CAM interventions is promising, but we need large scale trials with a substantial long-term follow-up to make evidence-based recommendations.

### Conclusions, challenges, and opportunities

The overview provides a clear picture of the limited and scattered evidence currently available for managing PCMP. Evaluations of psychological therapies are well-documented, but there is a continued need for high-quality evaluations of these therapies and a strong need for more high-quality evidence in the areas of pharmacological, physical therapy, and CAMS in order to formulate an evidence-based standard care plan for managing PCMP. It is also important to point out that most of the available evidence stems from either specific conditions, such as JIA, or samples including any chronic pain problem (i.e., including headaches, abdominal pain), with few studies focusing on pediatric musculoskeletal pain (MSK). Some progress in this area is being made in this area, by seeking evidence for children and adolescents with MSK pain in psychological and pharmacological trials. Beyond the lack of evidence, several challenges have been identified that present opportunities for future work in this area.

Intensive interdisciplinary treatment approaches (sometimes residential) have received preliminary support and are considered the gold standard for effective management of chronic pain in children and adolescents, including PCMP [56]. However, the availability of efficacy data for such intensive programs is limited. Furthermore, little is known about the cost-effectiveness of such intensive programs. A recent cost-analysis of one USA-based outpatient, intensive interdisciplinary program treating any type of pediatric chronic pain [57•] revealed a substantial reduction in outpatient visits, emergency department visits, and inpatient hospitalizations, which resulted in a cost saving for the hospital of 36,228 USD/patient on yearly basis. While this number does not include the costs for running the pain program, the authors advocated that these hospital cost savings would cover the cost associated with running the pain program [57•]. There is a crucial need for future studies to identify the effective components of these interdisciplinary packages for managing PCMP and their cost-effectiveness.

As highlighted earlier, one of the challenges to identify the effective components of interdisciplinary treatment is the historical exclusion of children and adolescents from randomized controlled trials (RCTs) evaluating pharmacological agents for chronic musculoskeletal pain. Beyond the ethical and practical challenges to implement such RCTs, research is largely dependent upon the interest and support from pharmaceutical companies to develop and evaluate the effectiveness of medication for PCMP. Unfortunately, this pharmaceutical interest and support is lacking, which represents a major barrier to overcome in this area. Other barriers, not exclusive to the evaluation pharmaceutical agents but applicable to all treatment components, include the need for better transparency of any study design, through registration of protocols and primary outcome measures as well as the underrepresentation of null findings in peer-reviewed publications. Such transparency would facilitate a comprehensive insight into the effective and active components of interdisciplinary treatment for PCMP. More systematic and standardized organization of data registries and controlled access for research purposes is one potential avenue to overcome these barriers and further this field.

An interesting area that has the potential to overcome the barrier of treatment accessibility in a cost-effective way is the role of remote treatment and how remote treatment options can be effectively integrated with face-to-face visits with the treatment team. While remote delivery (mainly including online delivery) of psychological treatment has grown in popularity and shown to be efficacious [26, 58••], evaluation of remote delivery of other treatment components (e.g., yoga instructions, lifestyle advice) and the integration of remote delivery with hospital appointments are lacking. The development and evaluation of online resources providing evidence-based support on a variety of treatment modalities, such as Teens Taking Charge (http://teens.aboutkidshealth.ca/sites/jia/en/Pages/default.aspx [30]), is growing, but continued evaluation of their effectiveness and integration within standard care plans will be crucial and has the potential to provide widespread, evidence-based care for PCMP.

Lastly, given the growing evidence on the crucial need for effective social support to be able to engage in daily activities despite experiencing PCMP, future investigations into (cost-)effective interventions to optimize peer and teacher support will be invaluable. Preliminary steps in this area are being made. For instance, a recent pilot peer mentorship program in which young adults with JIA provide mentorship to adolescents with JIA via Skype calls reported positive outcomes, with improvement in adolescent’s improved self-management skills and beliefs in their coping efforts being observed [37•]. Further challenges remain with regard to tailoring such programs to individual needs, as there was wide variation in the length and number of calls made and the program was only used amongst females [38]. Peer mentorship by another person living with the same condition can be valuable and facilitate more positive interactions with same-age pain-free peers. Furthermore, to the best of our knowledge, there are no standard guidelines available on how to support and integrate the child’s teacher within the treatment approach. The school environment is a paramount part of children’s life, which can be disrupted by the experience of PCMP. A better understanding of how to facilitate a supportive teacher-child relationship [59] and constructive teacher-parent relationship [60] would be imperative in overcoming this disruptive impact of PCMP on academic and social functioning.

To conclude, despite the relatively high prevalence of chronic musculoskeletal pain in youth, there is a sub-group of patients with severe needs who go untreated. There remains a lack of awareness of this problem, and so a lack of evidence for the efficacy or safety of most of our treatments. An absence of evidence is just that: a gap. In that gap, however, there is industry, creativity, and innovation. Although the development of pharmacological interventions has slowed, there is growth in the non-pharmacological field, including advances in psychological interventions. The optimal model for treatment remains an interdisciplinary programmatic approach in which different professionals combine treatment options in the service of the patient’s (or family) individual needs. However, delivery of services using this model and evaluation of these programs are rare. A positive first step for PCMP would be an interdisciplinary model in which one can bring together therapy and medical staff in a specific chronic pain clinic, and which utilizes online methods of increasing access to scarce expertise for this large population of patients.
